# Identification of Alleles in the MSP1 Gene Related to Complicated Malaria in Patients Infected with *Plasmodium falciparum* in Southeast of Iran

**Published:** 2019-06-24

**Authors:** Bentol Hoda Habibi-Shorkaei, Afsaneh Motevalli-Haghi, Mehdi Nateghpour, Leila Farivar, Homa Hajjaran, Soudabeh Etemadi

**Affiliations:** 1 Department of Parasitology and Mycology, School of Public Health, Tehran University of Medical Sciences, Tehran, Iran; 2 Center for Research of Endemic Parasites of Iran, Tehran University of Medical Sciences, Tehran, Iran

**Keywords:** *Plasmodium falciparum*, Sever malaria, Iran, PCR-RFLP

## Abstract

**Background:**

To overcome human malaria problem several solutions have been employed including extensive studies in the field of Plasmodia relevant antigens. The aim of this study was to determine allelic variation in the MSP1 gene of *Plasmodium falciparum* among some *falciparum* malaria-infected patients in Southeastern Iran.

**Methods:**

Twenty *P. falciparum* positive cases were enrolled from Sistan and Baluchistan Province, southeastern Iran in 2013–15. From each case, 1.5ml of peripheral blood was collected into EDTA contained tubes. Thick and thin blood smears were stained with standard Giemsa stain and were checked with conventional microscopical method. DNA was extracted from blood samples and amplification of block 2 MSP1 was performed using specific primers. Gel electrophoresis was done and results showed some amplification fragments corresponding to block 2 regions of Pf MSP1 gene. Finally, four samples from different allelic types were sent for sequencing process.

**Results:**

Fragments were different in size, so classified into six allelic types as kinds of 1–6 based on happening frequencies. Digestion of PCR products revealed two sub allelic types (A and B) within allelic types 2 and 3, but not in allelic types 1, 4, 5 and 6. Twenty percent of samples were sent for sequencing. Sequence alignment showed 78.95% to 91.83% identity between samples.

**Conclusion:**

Identity between samples and phylogenetic tree revealed that there is an extensive diversity range among isolates. Fifty percent of the isolates were under the risk of complicated malaria. Two of these patients (10%) needed special care and recovery was obtained after getting hospital services.

## Introduction

Malaria is an ancient problem created many calamities against human for many centuries. Parasites of the infection cause illness to 216 million persons and almost 445000 deaths per year. Pregnant women and children under 5yr old are two greatest groups damaged from this infection (1–3). The fearful condition of the disease in human is caused by *Plasmodium falciparum*. Cerebral malaria, blackwater fever, severe anemia and some other disorders occur in individuals who infected with *P. falciparum*. Such problems can result in death among the infected people if is not being treated with suitable drug.

To combat these conditions several malaria vaccines were produced and tested amongst some volunteer in clinical trials, but with poor consequences due to some reasons including gene diversities phenomenon in *P. falciparum* isolates. However, diversities in MSP1 gene demonstrate different alleles which such diversities can reveal different aspects of phenomena in *P. falciparum*. One of the most important aspects of the phenomena is emergence a number of complications in patients who infected with *P. falciparum* (4, 5).

Presence of some MSP1 sub allelic types have been seen in those individuals who infected with cerebral malaria. Studies of genetic diversity can provide valuable data in different aspects of *P. falciparum* malaria such as forecasting complicated cases in the infection. *Plasmodium falciparum* MSP1 gene produces a surface protein in merozoites in blood schizogony phase and it is a major candidate for providing a vaccine (6), but its notable diversity in different isolates is an obstacle within the progress. The gene produces a 190kDa glycoprotein including seven variable blocks putting at different places among five conserved and five semi-conserved blocks (5). Diversity recognition of the gene is important to determine alleles resulting severe malaria.

The aim of this study was to determine allelic variation in the MSP1 gene of *P. falciparum* among some malaria patients living in malarious areas of Sistan and Baluchistan Province of Iran in view of presence or absence of MSP1 alleles that cause cerebral malaria, using PCR-RFLP technique.

## Materials and Methods

### Study area

Sistan and Baluchistan Province is located in southeastern Iran. The latitude and longitude of the province are 27º0′ N and 62º0′ E respectively. The weather is warm and low humid in north of the province with average temperature of 31 °C, and warm and humid in south with average temperature of 30.2 °C. *Plasmodium vivax* and *P. falciparum* are prevalent Plasmodia species in the studied area. Moreover, *Anopheles culicifacies* and *An. stephensi* are the main vectors in southeastern Iran. The province is located on neighboring Pakistan and southwestern part of Afghanistan with 978 and 300km joint borderline respectively. Immigration load of Afghani and Pakistani population in the province is high with notable imported malaria infection. All the samples for this study were collected from those individuals who were living for long time in the studied areas.

### Blood samples

Twenty *P. falciparum* positive cases were enrolled between 2013 and 2015 for this study. From each case, 1.5ml of peripheral blood was collected into EDTA contained tubes. Previous to sampling an informed consent was prepared of the registered individuals.

### Detection of parasites

Thick and thin blood smears were stained with standard Giemsa stain and the slides were checked with conventional microscopical method with two expert microscopists. Mix infections were taken out from the study. Samples were stored at −20 °C until DNA extraction processes.

### DNA Extraction

DNA of *P. falciparum* was extracted from blood samples by using a high pure PCR template preparation kit (Roch REF: 11796828001) according to the manufacturer instructions.

### PCR-RFLP

PCR amplification of the block2 MSP1 was performed in 25µl reaction that containing, 10 µl Distilled water, 10µl master mix, 3ng DNA, 1µl forward primer, 1µl revers primer. Designing primers and PCR condition were performed (7).

The sets of following primers were used for amplification: Forward 5′CTAGAAGCTTTA GAAGATGCAGTATTG3′ Revers 5′GTACG TCTAATTCATTTGCACG AAT3′ PCR amplification was carried out using initial denaturation about 5min in 95 °C, 30 cycles of 1 min at 94 °C, 2min in 57 °C, 2min in 72 °C and final extension 5min in 72 °C in a thermal cycler. Four µl of PCR product was run on 1.5% agarose gel with simply safe (EUREx, Cat: E 4600-01). Finally the process was visualized with gel duct and resulted in some photographs.

### RFLP (Restriction Fragment Length Polymorphism)

Product amplification of MSP1 gene block 2 zones was digested with Dra-1 endonuclease with active site of TTT AAA (Cat: FD0224). The digestion process was carried out in a 30 µl composition of 17µl distilled water, 2µl 10 X fast digest buffer, 10µl PCR product and 1 µl fast digest enzyme. The compound was incubated at 37 °C for 5min and the incubation was continued at 65 °C for 5min to inactive the enzyme. Overall 18µl of the digested PCR product was analyzed on a 3% agarose gel and then stained with simply safe stain. Visualizing process was conducted as mentioned previously.

### Sequencing

Four PCR products were selected for sequence processes and analyzed with ClustalW2 and Blast software.

## Results

**Fig. 1.**
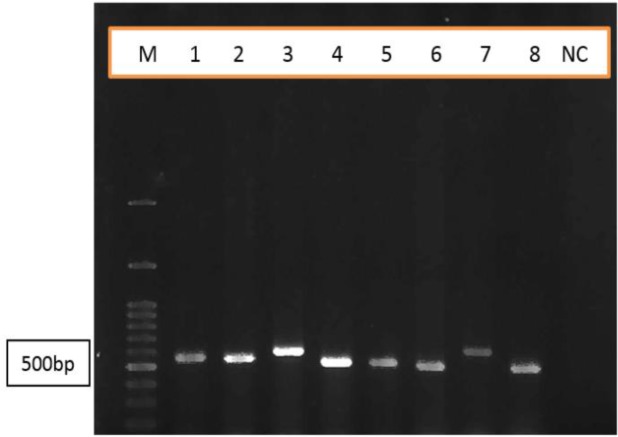
PCR product of MSP1 gene (block 2) of *Plasmodium falciparum* Eight isolates were visualized with 2% agarose gel and stained with simply safe. M= DNA size Marker; lane1–8 isolates, NC= negative control

Genetic diversity of *P. falciparum* block2 MSP1 gene was considered from clinical isolates collected from Sistan and Baluchistan Province in southeastern Iran (Table 1). All of the 20 isolates showed gene amplified expression in block 2 regions of MSP1 gene. Variation of amplified products was identified from 480 to 600bp (Fig. 1). The isolates were classified into six allelic types as kinds of 1–6 based on happening frequencies. Type 1 possessed the highest frequency (50%) and allelic types 4, 5, 6 the lowest frequency (5%) (Table 2).

**Table 1.** Demographic data of patients based on nationality, gender and age

**Nationality**	**Gender**		**Age (yr)**			**Total**

	**Male**	**Female**	**< 20**	**20–40**	**> 40**	
**Iranian**	10	4	3	9	2	14
**Afghan**	2	2	1	2	1	4
**Pakistani**	0	2	1	1	0	2
**Total**	12	8	5	12	3	20

**Table 2.** Allelic types of MSP1 (Block2) of *Plasmodium falciparum* isolates based on molecular weight of PCR products (n= 20)

**Size of PCR product**	**Allelic type %**	**No of *P. falciparum* isolate**	**Allelic frequency %**
**500bp**	1	10	50
**550bp**	2	5	25
**600bp**	3	2	10
**480bp**	4	1	5
**570bp**	5	1	5
**580bp**	6	1	5

**Fig. 2.**
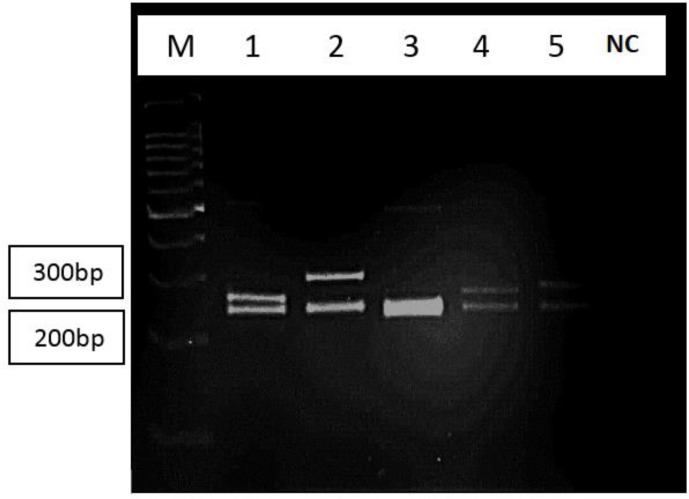
RFLP pattern of MSP1 gene block 2(digested with Dra-1) *Plasmodium falciparum* isolates. Digested products were resolved on 4% agarose gel. Lane M-DNA size marker, lane1 type 4 allele, lane 2 type 3 allele (3A sub allel), lane 3 type 1 allele, lane 4 type 5 allele, lane 5 type 6 allele, NC: negative control.

PCR products were digested with Dra-1 restriction enzyme. RFLP identified the subtypes of allelic types among the isolates. Digestion of PCR products revealed two sub allelic (types A and B) within allelic types 2 and 3. While allelic types 1, 4, 5, 6 did not show any subtype (Table 3, Fig. 2). The phylogenetic tree showed a wide diversity among the studied samples which some of them had considerable homology with 3D7 isolate and some others had more homology with HN2 China isolate. The Belem strain of *P. vivax* was used as outgroup for this analysis (Table 4).

**Table 3.** Allelic and sub allelic types of MSP1 (block2) of *Plasmodium falciparum* isolates on the basis of PCR-RFLP (digested with Dra-1 restriction enzyme) (n= 20)

**Allelic type**	**Size of PCR product**	**No of isolates**	**Sub allelic type**	**Size of digested fragment**	**No of isolate**	**Frequency %**
**1**	500	10/20=50%	---------	250,250	10/10	100
**2**	550	5/20=25%	2A	270,280	3/5	60
			2B	250,300	2/5	40
**3**	600	2/20=10%	3A	280,320	1/2	50
			3B	250,350	1/2	50
**4**	480	1/20=5%	--------	250,230	1/1	100
**5**	570	1/20=5%	--------	280,290	1/1	100
**6**	580	1/20=5%	--------	280,300	1/1	100

**Table 4.** Percent Identity among isolates collected from Iran (Accession number: KY026097.1, KYO26098.1, KY026095.1, KY026096.1) and *Plasmodium falciparum* 3D7 (Accession number XM_001352134.1) and HN2 isolate from China (Accession number AF062349.1). *Plasmodium* *vivax* Belem isolate used an out group

**1**	**2**	**3**	**4**	**5**	**6**	**7**
**vivax**	3 D7	If 77a	If 140	If 133	If 95a	china
**100.00**	43.69	43.54	43.74	40.41	39.28	41.36
**43.69**	100.00	90.26	92.20	78.21	79.71	83.63
**43.54**	90.26	100.00	91.83	78.95	79.15	78.85
**43.74**	92.20	91.83	100.00	80.38	79.75	79.27
**40.41**	78.21	78.95	80.38	100.00	90.37	91.10
**39.28**	79.71	79.15	79.75	90.37	100.00	91.60
**41.36**	83.63	78.85	79.27	91.10	91.60	100.00

**Fig. 3.**
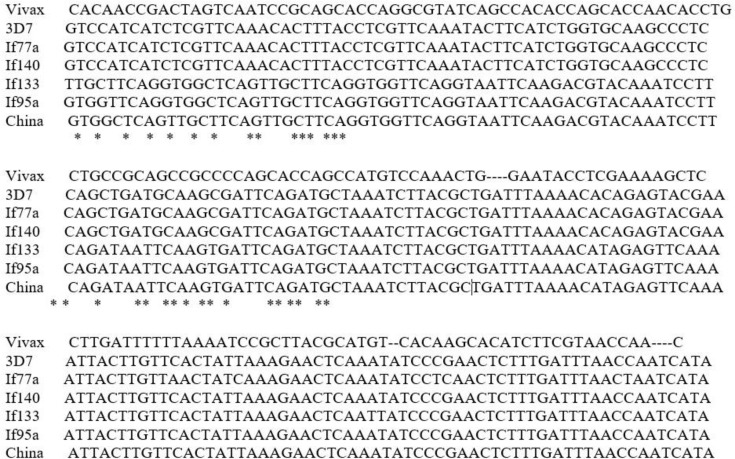
Partial nucleotide sequences alignment of *P.f.* MSP1 gene in *Plasmodium falciparum* isolated collected from malarious areas of Iran and were compared to the genes of China (HN2), vivax (Belem) and 3D7 reference strains.

**Fig. 4.**
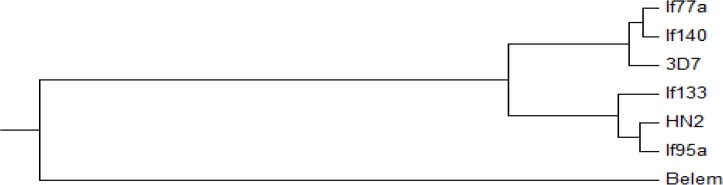
Phylogenetic tree between isolates collected from southeastern Iran (Accession number: KY026097.1, KYO26098.1, KY026095.1, KY026096.1) and *Plasmodium falciparum* 3D7 (Accession number XM_001352134.1) and CHINA HN2 (Accession number AF062349.1). *Plasmodium vivax* Belem isolate used an out group.

## Discussion

Investigations about genetic diversities in parasite population can prepare much information in the field of geographical distribution, genomic markers, clinical manifestation, drug resistance and gene polymorphism for relevant researchers (8–12). Genetic diversity plays a crucial role in the natural acquisition of immunity in malaria disease and it is a major problem in the pathway of control strategies to prevent the distribution of the infection. Combating drug resistance in malaria parasites or producing potential vaccines require comprehension of population structure of the parasite (13). For this purpose, a lot of studies have been conducted by researchers to access genomic markers and polymorphism of the parasite. Among the studies about diversity of *Pf*MSP1 gene, a few of them have been focused on relationship between the gene and probable malignancy of *P. falciparum* (14). There is a significant correlation between diversity in MSP1 gene and complicated cases of malaria, and in contrast to them, some others mentioned there is no association among them (15–18, 7, 10).

Obtaining different results about the mentioned subject are due to the different employed methods to found diversity in this gene. For example nested PCR was used to found frequency of K1, Mad20 and Ro33 alleles in the parasite population (18–20). However, PCR-RFLP was used to report diversity in the gene and reported five allelic types. Nine different alleles were reported from of 67 isolates and mentioned MSP1 gene in *P. falciparum* have shown an extended variation in size and pattern (19). Any correlation was mentioned between these alleles and malignancy in *P. falciparum*. There was no significant relationship between genetic diversity of MSP1 and severity in malaria, however, Mad20 allelic type increased with the severity of malaria. On the other hand, there are a few alleles in *Pf* MSP1 gene which are more predominant in cerebral malaria when they used PCR-RFLP to investigate diversity in the gene. Five allelic types including eight genetic types were identified, they implied that sub allelic types 2A and 3A with 300–200 and 280–200bp were more prevalence in patients got involved with cerebral malaria these sub allelic types were recognized when they used PCR-RFLP with Dra-1 restriction enzyme. Moreover, sub allelic 2B with 250–25bp was seen in both cerebral and non-cerebral malaria cases. Presence of certain alleles may cause severe disease in patients.

In the present study six allelic types were classified by PCR-RFLP, also two sub allelic types were identified when PCR products digested with Dra-1, results showed that there is a large diversity in the weight of the products fragments when compared with Farooq et al results (17). So, diversity of the gene is high in various areas. Anyway, 65% of the patients who registered in the present study showed allelic types like reported by Farooq and 50% were under the risk of complicated malaria (17). Two of these patients (10%) needed special care and recovery was obtained after getting hospital services. Isolating the genes that involve in producing cerebral malaria provides a valuable opportunity for prevention of deleterious complications of *falciparum* malaria in endemic areas.

Moreover, sequence alignment among the samples showed 78.95% to 91.83% identity. There is an extension of diversity among the samples. Phylogenetic tree revealed that there is an extensive diversity range between isolates so that some of the isolates achieved in the present study showed a close relationship with isolate HN2 from China and the others have this association *P. falciparum* 3D7. There is high diversity in the gene in the specific population of the parasite.

## Conclusion

MSP1 gene of *P. falciparum* in Iranian isolates showed an extensive diversity, moreover, potential characteristics to produce sever malaria particularity cerebral malaria is a burglar alarm for the health authorities to provide accessible facilities for residents in malaria endemic area.
